# Clinical profiles and quality of care of subjects with type 2 diabetes according to their cardiovascular risk: an observational, retrospective study

**DOI:** 10.1186/s12933-021-01251-4

**Published:** 2021-03-06

**Authors:** Basilio Pintaudi, Alessia Scatena, Gabriella Piscitelli, Vera Frison, Salvatore Corrao, Valeria Manicardi, Giusi Graziano, Maria Chiara Rossi, Marco Gallo, Domenico Mannino, Paolo Di Bartolo, Antonio Nicolucci

**Affiliations:** 1 Diabetes Unit, Niguarda Cà Granda Hospital, Milan, Italy; 2 San Donato Hospital, Arezzo, Italy; 3 Diabetology, ASST Nord Milan, Sesto San Giovanni, Italy; 4 Internal Medicine and Diabetology Service, ULSS6 Cittadella, Italy; 5 Department of Internal Medicine, ARNAS Civico Benfratelli Hospital, University of Palermo, Palermo, Italy; 6 Diabetes Clinic, Azienda USL-IRCCS Di Reggio Emilia, Reggio Emilia, Italy; 7 Center for Outcomes Research and Clinical Epidemiology, CORESEARCH, Corso Umberto I, 65122 Pescara, Italy; 8 AOU Città Della Salute E Della Scienza, Presidio Ospedaliero Molinette, Torino, Italy; 9 Endocrinology and Metabolic Unit, GOM, Reggio Calabria, Italy; 10 AUSL Diabetes Unit Romagna, Ravenna, Italy

**Keywords:** Cardiovascular risk, Type 2 diabetes, Quality of care

## Abstract

**Background:**

The European Society of Cardiology (ESC) recently defined cardiovascular risk classes for subjects with diabetes. Aim of this study was to explore the distribution of subjects with type 2 diabetes (T2D) by cardiovascular risk groups according to the ESC classification and to describe the quality indicators of care, with particular regard to cardiovascular risk factors.

**Methods:**

The study is based on data extracted from electronic medical records of patients treated at the 258 Italian diabetes centers participating in the AMD Annals initiative. Patients with T2D were stratified by cardiovascular risk. General descriptive indicators, measures of intermediate outcomes, intensity/appropriateness of pharmacological treatment for diabetes and cardiovascular risk factors, presence of other complications and overall quality of care were evaluated.

**Results:**

Overall, 473,740 subjects with type 2 diabetes (78.5% at very high cardiovascular risk, 20.9% at high risk and 0.6% at moderate risk) were evaluated. Among people with T2D at very high risk: 26.4% had retinopathy, 39.5% had albuminuria, 18.7% had a previous major cardiovascular event, 39.0% had organ damage, 89.1% had three or more risk factors. The use of DPP4-i markedly increased as cardiovascular risk increased. The prescription of secretagogues also increased and that of GLP1-RAs tended to increase. The use of SGLT2-i was still limited, and only slightly higher in subjects with very high cardiovascular risk. The overall quality of care, as summarized by the Q score, tended to be lower as the level of cardiovascular risk increased.

**Conclusions:**

A large proportion of subjects with T2D is at high or very high risk. Glucose-lowering drug therapies seem not to be adequately used with respect to their potential advantages in terms of cardiovascular risk reduction. Several actions are necessary to improve the quality of care.

## Background

Type 2 diabetes (T2D) is associated with increased cardiovascular morbidity and mortality [1]. Patients with T2D have a significant increase in the risk of coronary heart disease and ischemic stroke and a 1.5 to 3.6 fold increase in mortality [1]. Diabetes is also an important risk factor for heart failure, peripheral artery disease, and microvascular complications, negatively impacting quality of life and life expectancy. It is estimated that patients with diabetes have a reduction in life expectancy of about 4–8 years, compared to individuals without diabetes [2, 3]. Although great advances in prevention and therapy have resulted in significant reductions in diabetes-related cardiovascular mortality, cardiovascular risk still remains high in most patients with diabetes. Considering the growing number of survivors of cardiovascular events and the global diabetes epidemic, the number of T2D patients at higher cardiovascular risk is expected to increase, this posing a major challenge to healthcare systems. Therefore, the implementation of cost-effective strategies for cardiovascular risk reduction in this population is urgent [4]. Recent evidence indicates that the cardiovascular risk in T2D is highly heterogeneous, being it not universally similar to the risk of patients with previous cardiovascular disease [5–8].

The stratification of people with diabetes into different groups at different cardiovascular risk allows the recognition of those who could benefit most from more intensive cardiovascular prevention. Therefore, it may be useful to develop rational strategies to more intensively detect and treat patients at higher risk, while it may be reasonable and economically sound to use less intensive therapeutic approaches in those at lower cardiovascular risk.

Recently, the guidelines of the European Society of Cardiology (ESC) in collaboration with the European Society for the Study of Diabetes (EASD) [9] proposed a stratification of cardiovascular risk among people with diabetes based on the presence of established atherosclerotic disease, organ damage (proteinuria, eGFR < 30 ml/min/1.73m^2^, left ventricular hypertrophy, retinopathy), or multiple risk factors (age, smoking, obesity, hypertension, dyslipidemia). This stratification subdivides people with diabetes into three groups: very high risk (10-year event risk > 10%), high risk (between 5 and 10%), and moderate risk (< 5%). The proposed stratification has important implications both on the therapeutic targets to be reached and the choice of treatment.

In light of this new classification, aim of this study was to explore the distribution and clinical characteristics of subjects with T2D cared for by Italian diabetes centers according to cardiovascular risk groups and to describe quality of care indicators, with particular reference to cardiovascular risk factors.

## Methods

This was an observational, retrospective study including a large sample of patients cared for by 258 Italian diabetes clinics participating in the Annals initiative of the Italian Association of Clinical Diabetologists [Associazione Medici Diabetologi (AMD)] during 2018. AMD Annals is a monitoring and continuous quality of care improvement national initiative. This initiative is based on clinical data stored in electronic medical records of a large network of diabetes clinics, which are periodically extracted and used to assess specific quality indicators of diabetes care. All participating diabetes centers obtained the authorization of local Ethics Committees. A software was developed to enable the extraction of the information needed from electronic medical record systems used for the everyday management of outpatients [10]. Patients with type 2 diabetes were stratified on the base of their cardiovascular risk, according to the recent European Society of Cardiology (ESC) guidelines [9]. General descriptive indicators, intermediate outcome measures regarding intensity and appropriateness of diabetes pharmacological therapy, cardiovascular risk factors, chronic complications, and overall health care quality were evaluated. The denominator of each indicator was represented by patients with at least one detection of the parameters during the index year. When more than one measurement for the same patient in the index year was available, the more recent value was considered.

Overall quality of health care was evaluated by the Q-Score [11–13], calculated from process and intermediate outcome measures of HbA1c, blood pressure, LDL cholesterol and microalbuminuria (detection in the last 12 months, achievement of specific targets and adequate therapy prescription). For each patient a score between 0 and 40 was calculated as increasing index of good quality of care. The Q-Score showed to predict the incidence of major cardiovascular events in previous studies. In particular, the three-year risk of developing a cardiovascular event was 80% higher in subjects with a score < 15 and more than 20% higher in subjects with a score between 20 and 25, as compared to subjects with a score > 25 [11, 12]. Furthermore, the Q-Score predicted the variability of key risk factors for complications in Type 2 Diabetes [13].

Socio-demographic and clinical characteristics as well as health care quality indicators were expressed as mean and standard deviation or percentage and compared between the different risk classes with ANOVA or chi-square, for continuous or categorical variables, respectively. The results were calculated on non-missing values, without any imputation technique. A p value < 0.05 was considered as statistically significant. All analyses were conducted with SAS software (version 9.4).

## Results

Overall, 473.740 patients with type 2 diabetes were evaluated. When subjects were stratified according to their cardiovascular risk, 78.5% of them were at very high risk, 20.9% at high risk and 0.6% at moderate risk. Patients characteristics by the different risk classes are reported in Table 1. The class of moderate risk was small. It included subjects with a low mean age and a short diabetes duration; a quarter of subjects were newly-diagnosed; BMI levels were significantly lower than the other risk classes. No gender difference between the cardiovascular risk classes was detected. Among high-risk subjects, 44.7% had at least a ten years diabetes duration. Among subjects at very high risk, 18.7% had a history of major cardiovascular event, 26.4% had retinopathy, 39.5% albuminuria and 8.8% eGFR < 30 ml/min/m^2^, and 89.1% had three or more cardiovascular risk factors. With regard to anti-hyperglycemic drugs, the use of DPP4-inhibitors increased with increasing cardiovascular risk; for secretagogues the same trend was documented. A linear increase, even though within low percentages, was observed also for GLP1-RAs. The use of SGLT2 inhibitors was also limited and only slightly higher in very high risk subjects. As expected, the highest percentage of subjects treated with insulin was detected in the very high risk group.

**Table 1:** Characteristics of type 2 diabetes population according to cardiovascular risk

	Moderate risk	High risk	Very high risk	p
Number of subjects	2,819	98,781	372,140	
Age (years)	41.6 ± 6.8	66.7 ± 13.1	70.0 ± 10.2	< 0.0001
Sex (% male)	57.6	56.5	57.2	0.0003
New diagnoses (%)	25.3	9.9	4.9	< 0.0001
Diabetes duration (years)	3.0 ± 2.8	10.1 ± 8.9	12.9 ± 9.5	< 0.0001
BMI (kg/m^2^)	25.6 ± 2.8	27.0 ± 4.5	30.0 ± 5.5	< 0.0001
Smokers (%)	0.0	7.6	19.2	< 0.0001
HbA1c (%)	7.3 ± 1.7	7.1 ± 1.3	7.2 ± 1.2	0.002
Blood pressure (mmHg)				
Systolic	116.4 ± 11.2	130.1 ± 17.0	136.5 ± 18.3	< 0.0001
Dyastolic	73.3 ± 7.7	75.9 ± 9.2	76.9 ± 9.7	< 0.0001
Total Cholesterol (mg/dl)	175.1 ± 31.2	174.0 ± 33.9	167.1 ± 38.8	< 0.0001
LDL Cholesterol (mg/dl)	97.8 ± 20.6	97.2 ± 27.8	91.0 ± 32.7	< 0.0001
HDL Cholesterol(mg/dl)	48.5 ± 13.2	51.1 ± 13.7	48.1 ± 12.7	< 0.0001
Triglicerides (mg/dl)	135.8 ± 106.0	125.8 ± 78.1	141.1 ± 81.6	< 0.0001
Albuminuria (%)	0.0	0.0	39.5	< 0.0001
eGFR < 30 ml/min/1,73m^2^ (%)	0.0	0.0	8.8	< 0.0001
Anti-hyperglycemic therapy (%)				
Metformin	72.0	71.1	69.0	< 0.0001
DPP4-i	13.3	19.8	21.5	< 0.0001
Secretagogues	7.7	15.9	16.3	< 0.0001
Glinides	1.2	3.6	3.7	< 0.0001
SGLT2-i	8.5	7.7	10.0	< 0.0001
Glitazones	3.1	4.0	4.4	< 0.0001
Acarbose	1.1	2.0	2.5	< 0.0001
GLP1-RA	2.4	3.9	6.4	< 0.0001
Insulin	27.6	24.4	34.5	< 0.0001
Diabetes treatment scheme (%)				
Oral Monotherapy	38.1	35.7	27.4	< 0.0001
Oral Two fold therapy	19.2	23.0	21.7	< 0.0001
Three or more oral drugs	3.3	6.0	6.5	< 0.0001
GLP1-RA ± other drugs	2.4	3.9	6.4	< 0.0001
Insulin + oral drugs	12.8	12.8	19.1	< 0.0001
Multiple daily insulin injections	14.5	10.9	14.2	< 0.0001
Anti-hypertensive therapy (%)	0.0	32.9	80.4	< 0.0001
Lipid-lowering therapy (%)	0.0	18.0	72.6	< 0.0001
Retinopathy (%)	0.0	0.0	26.4	< 0.0001
Non proliferative	0.0	0.0	19.5	
Pre-proliferative	0.0	0.0	1.9	
Proliferative	0.0	0.0	1.8	
Laser-treated	0.0	0.0	2.6	
Oftalmopathy	0.0	0.0	0.2	
Blindness	0.0	0.0	0.3	
Previous myocardial infarction (%)	0.0	0.0	9.5	< 0.0001
Previous stroke (%)	0.0	0.0	3.3	< 0.0001
Established cardiovascular disease (%)	0.0	0.0	18.7	< 0.0001
Dialisys (%)	0.0	0.0	0.3	< 0.0001

Health care quality indicators are reported in Table 2. In the very high risk class, the percentage of patients with HbA1c > 8.0% was lower and the percentage of patients with LDL < 100 mg/dl was higher than the other classes. Conversely, in this class the percentage of patients with high blood pressure values and the percentage of obese patients were higher compared with the other classes.

**Table 2:** Health care quality indicators according to cardiovascular risk

	Moderate risk	High risk	Very high risk	p
HbA1c ≤ 7.0% (%)	57.4	58.0	51.6	< 0.0001
HbA1c > 8.0% (%)	22.3	16.1	18.4	< 0.0001
Blood pressure ≥ 140/90 mmHg (%)	0.0	31.0	50.8	< 0.0001
LDL Cholesterol < 100 mg/dl (%)	49.8	54.7	65.6	< 0.0001
LDL Cholesterol ≥ 130 mg/dl (%)	0.0	9.1	13.1	< 0.0001
Subjects with HbA1c ≤ 7.0%, with C-LDL < 100 mg/dl and with BP < 140/90 mmHg (%)	31.0	24.3	18.6	< 0.0001
BMI ≥ 30 kg/m^2^(%)	0.0	14.7	46.6	< 0.0001
Subjects not treated with insulin despite HbA1c ≥ 9.0% (%)^a^	39.2	38.4	25.5	< 0.0001
Subjects with HbA1c ≥ 9.0% despite insulin therapy (%)^b^	31.6	18.2	15.7	< 0.0001
Subjects not treated with anti-hypertensive despite BP ≥ 140/90 mmHg (%)^c^	n.a	48.2	22.5	< 0.0001
Subjects with BP ≥ 140/90 mmHg despite anti-hypertensive therapy (%)^d^	n.a	46.8	48.8	< 0.0001
Subjects not treated with lipid-lowering drugs despite C-LDL ≥ 130 mg/dl (%)^e^	n.a	60.1	45.5	< 0.0001
Subjects with C-LDL ≥ 130 mg/dl despite lipid-lowering therapy (%)^f^	n.a	16.9	9.7	< 0.0001
Subjects with a previous CV event (heart attack and/or stroke) treated with antiplatelet therapy (%)	n.a	n.a	76.1	-
Mean Q Score	32.2 ± 6.8	30.1 ± 7.6	28.2 ± 8.3	< 0.0001
Subjects with Q Score < 15 (%)	0.0	1.6	4.1	< 0.0001
Subjects with Q Score > 25 (%)	74.0	66.1	59.4	< 0.0001

^a^Denominator: all the subjects with HbA1c ≥ 9.0%

^b^Denominator: all the subjects treated with insulin

^c^Denominator: all the subjects with BP ≥ 140/90 mmHg

^d^Denominator: all the patients treated with anti-hypertensive drugs

^e^Denominator: all the subjects with C-LDL ≥ 130 mg/dl

^f^Denominator: all the subjects treated with lipid-lowering drugs

*n.a.* notapplicable

Indicators of therapeutic intensity reiterated greater attention to subjects at higher risk, except for the very high percentage of patients with inadequate blood pressure values despite antihypertensive therapy.

Q-score data documented lower mean scores in the highest cardiovascular risk classes. The percentage of subjects with a Q-score < 15 was very low in all groups.

## Discussion

Diabetes has long been considered an equivalent of cardiovascular risk. This claim was based on the results of a Finnish study [14], in which patients with T2D without coronary artery disease (CHD) had a coronary artery mortality rate similar to that of subjects without diabetes who had had a previous coronary event. Diabetes also increases coronary death rates due to the worse prognosis after having the first event of coronary heart disease. These arguments have led in the past to recommend that diabetic patients should be treated as a separate high-risk category, without the need for stratification [15].

However, recent evidence indicates that the risk of CHD in T2D is not universally similar to the risk of patients with previous cardiovascular disease, but is highly heterogeneous. A meta-analysis of 13 epidemiological studies, including 45.108 patients with and without diabetes, found that in T2D patients without prior CHD the risk of CHD was 43% lower than individuals without diabetes with a prior myocardial infarction [5]. In a large population cohort [6] that included 1.586.061 adults aged 30 to 90 years followed for 10 years, the risk of coronary artery disease was much lower among subjects with T2D without CHD than in patients with CHD without diabetes, compared to patients without neither CHD, nor T2D [HR: 1.70 (95% CI 1.66–1.74) vs. 2.80 (95% CI 2.70–2.85), respectively]. In another meta-analysis of observational studies in patients with DM2 [7], cardiovascular risk was assessed by the coronary calcium score at baseline. The Authors found a prevalence of 28.5% of patients with a calcium score of zero, indicating a 5-year survival rate similar to that of patients without diabetes [8]. Therefore, a subgroup with lower CHD risk is likely to exist, particularly including patients younger than 40 years with a short disease duration.

In line with these findings, we found that a large proportion of patients show a 10-year risk of major cardiovascular events over 10%, due to the presence of a previous cardiovascular event, organ damage, or multiple cardiovascular risk factors. However, a small percentage of the study population could be considered ad intermediate risk (i.e. below 5%), thus confirming the existence of a continuum in cardiovascular risk among people with T2D. Furthermore, our study population was represented by patients attending diabetes clinics; as such, the proportion of individuals with moderate risk was presumably underestimated. Also, in our study population the prevalence of established cardiovascular disease was 18.7%, lower than the 30% European prevalence among patients with T2D reported by Einarson et al. [16]. However, in the same paper, the prevalence of CVD disease in Italy was reported to be 14.8%.

Once it is clear that the cardiovascular risk profile is heterogeneous, it is necessary to focus on the most advantageous and appropriate therapeutic strategy for each risk class. Our data showed that, after metformin and insulin, the use of DPP4-i drugs is prevalent. This pharmacological class has shown neutral effects from the cardiovascular point of view for almost all the molecules of the class [17]. Their use in the highest risk class does not appear completely justified when compared with the underutilization of some pharmacological classes with proven cardiovascular benefit (i.e. GLP1-RA and SGLT2-i) [18–21].

The mechanisms underlying the drug-related reduction of cardiovascular outcomes associated with the use of SGLT2-i and GLP1-RA are still under investigation [22]; however, their clinical utility goes uncontested [23]. In addition to the direct effect of these drugs on the cardiovascular system, optimal glycemic control remains essential [24].

The wide use of metformin in our study population is also justified by existing evidence. In particular, metformin use following acute myocardial infarction was associated with a reduction in risk of all-cause mortality [25]. On the other hand, a significant use of secretagogues and glinides in the classes with higher cardiovascular risk was documented. The use of these drugs raises concerns in particular due to the risk of hypoglycemic episodes and it has recently been associated with an increase in all-cause mortality [26].

To our knowledge, this is the first study investigating the clinical aspects and quality of care indicators in subjects with T2D according to the stratification of cardiovascular risk proposed by ESC. We have described detailed clinical characteristics of people attending Italian diabetes centers, thus allowing the identification of patients needing a more intensive care. The other novelty of our study consists in the assessment of health care quality indicators according to cardiovascular risk. This represents a relevant aspect to improve the level of care, overcome clinical inertia, and implement cost-effective strategies for cardiovascular risk reduction. The implications of the findings of our study in the context of existing research are important. The large sample of patients studied allowed to consistently define the clinical characteristics of patients with diabetes belonging to the different cardiovascular risk classes. This could generate further studies useful for intercepting risk trajectories, in particular by studying the predictive factors for the transition of lower-risk subjects towards higher risk profiles. In this way, it will be possible to act preventively on the categories of the most vulnerable subjects and those with a higher likelihood of worsening their cardiovascular risk.

Our findings also underline the importance of a deeper involvement of cardiologists in the management of T2D and established cardiovascular disease, to ensure that cardio-protective therapies are used along with other evidence-based therapies to improve patient outcomes [27, 28].

Our study has limitations. First, data analyzed refer to 2018; however, it is unlikely that the characteristics of patients attending diabetes clinics have changed in the last two years. On the other hand, rates of use of the different antihyperglycemic classes may have changed in most recent years, in the light of the accumulation of a large body of evidence supporting the positive cardio-renal effects of SGLT2i and GLP1-RA. Finally, data on hospitalizations and hypoglycemic episodes were not available; however, the history of major cardiovascular events was derived directly from electronic medical records.

## Conclusions

Our study showed that the majority of individuals with T2D has a very high cardiovascular risk. We were able to define the features of the subjects at moderate risk. It included subjects with a very low mean age, a short diabetes duration and a low BMI. The analysis of pharmacological therapies showed an unexpected underuse of the classes of anti-hyperglycemic drugs that can offer cardiovascular protection, particularly GLP1-RA and SGLT2-i.

The assessment of the level of quality of care showed different areas of intervention on which to target therapeutic and preventive action. Intervention on modifiable risk factors such as BMI and smoking should be carefully considered during clinical practice. The finding of a large percentage of subjects with high blood pressure values in the very high risk class requires a reflection on the role of the diabetes specialist in the management of cardiovascular risk factors. Risk stratification can help healthcare professionals to better personalize the care of different types of patients. Specifically, the use of anti-hyperglycemic drugs with documented positive cardiovascular effects is desirable. The stratification could also raise awareness among stakeholders on the identification of more efficient cardiovascular diagnostic-therapeutic pathways. Further studies are needed to test the effect of differentiated care processes on different risk categories from both a clinical and an economic point of view.

## Data Availability

The datasets used and/or analyzed during the current study are available from the corresponding author on reasonable request.

## Abbreviations

T2D: Type 2 Diabetes
DPP4-i: Dipeptidil Peptidasi 4- inhibitor
GLP1-RA: Glucagon-Like Peptide 1—Receptor Agonist
SGLT2-i: Sodium-GLucose co-Transporter-2 inhibitors
BMI: Body Mass Index
